# Ferroelectric Polymers Exhibiting Negative Longitudinal Piezoelectric Coefficient: Progress and Prospects

**DOI:** 10.1002/advs.201902468

**Published:** 2020-02-05

**Authors:** Yang Liu, Qing Wang

**Affiliations:** ^1^ Department of Materials Science and Engineering The Pennsylvania State University University Park PA 16802 USA

**Keywords:** electrostriction, ferroelectric polymers, morphotropic phase boundary, negative longitudinal piezoelectric coefficient, piezoelectricity

## Abstract

Piezoelectric polymers are well‐recognized to hold great promise for a wide range of flexible, wearable, and biocompatible applications. Among the known piezoelectric polymers, ferroelectric polymers represented by poly(vinylidene fluoride) and its copolymer poly(vinylidene fluoride‐*co*‐trifluoroethylene) possess the best piezoelectric coefficients. However, the physical origin of negative longitudinal piezoelectric coefficients occurring in the polymers remains elusive. To address this long‐standing challenge, several theoretical models proposed over the past decades, which are controversial in nature, have been revisited and reviewed. It is concluded that negative longitudinal piezoelectric coefficients arise from the negative longitudinal electrostriction in the crystalline domain of the polymers, independent of amorphous and crystalline‐amorphous interfacial regions. The crystalline origin of piezoelectricity offers unprecedented opportunities to improve electromechanical properties of polymers via structural engineering, i.e., design of morphotropic phase boundaries in ferroelectric polymers.

## Graphical abstract

Negative longitudinal piezoelectric coefficients in ferroelectric polymers are determined to arise from the negative longitudinal electrostriction in the crystalline domain of polymers, independent of amorphous and crystalline–amorphous interfacial regions. The crystalline origin of piezoelectricity offers unprecedented opportunities to improve the electromechanical properties of polymers via the design of morphotropic phase boundaries in ferroelectric polymers.

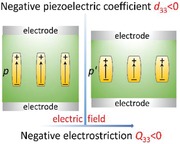

## 1 Introduction

**Figure 1:**
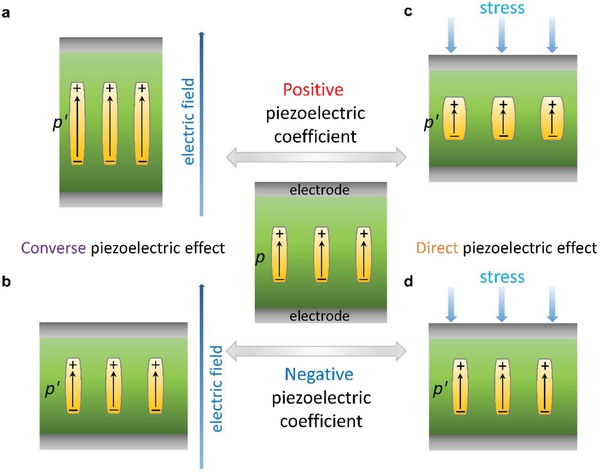
a,b) Schematic of converse piezoelectric effect in response to an external electric field in PZT and PVDF. c,d) Schematic of direct piezoelectric effect response to an external stress field in PZT and PVDF. For the case of PVDF with negative piezoelectric coefficient in (b) and (d), the dipoles are assumed to be rigid (*p* = *p*′, *p* is the dipole moment) according the dimensional model,^36^, ^37^ which differs from that in PZT with positive piezoelectric coefficient in (a) and (c). In the electrostriction model, the dipoles shrink rather than expand along the field (*p* < *p*′) corresponding to a negative sign, which is responsible for the negative piezoelectric coefficient in PVDF and P(VDF‐TrFE)s.

Piezoelectricity refers to the conversion of electrical to mechanical energies and vice versa, enabling a wide range of applications in the fields of industrial automation, medical diagnostics, electronics, and defense industry.^1^, ^2^ Semicrystalline poly(vinylidene fluoride) (PVDF) is the first piezoelectric polymer discovered by Kawai in 1969.^3^ Unusually, PVDF and its copolymer poly(vinylidene fluoride‐*co*‐trifluoroethylene) (P(VDF‐TrFE)) exhibits negative longitudinal piezoelectric coefficients.^4^, ^5^, ^6^, ^7^, ^8^, ^9^, ^10^, ^11^, ^12^, ^13^ Under the usual conditions, applying an electric field along the polarization direction leads to expansion of piezoelectrics such as lead zirconate titanate (PZT) ceramics (**Figure** **1**a) with positive longitudinal piezoelectric coefficients (*d*_33_ > 0). By contrast, PVDF with a negative longitudinal piezoelectric coefficient (*d*_33_ < 0) contracts in the direction of the applied electric field (its direction remains the same as polarization's direction) when an electric field is turned on (Figure 1b), and expands when the field is switched off.

PVDF and P(VDF‐TrFE)s exhibit the strongest piezoelectric responses (*d*_33_ ≈ −30 pC N^−1^) among the polymer materials.^4^, ^5^, ^6^, ^7^, ^8^, ^9^, ^10^, ^11^, ^12^, ^13^ While the introduction of voids into polymers leads to formation of cellular and porous polymer electrets which may exhibit much higher *d*_33_ values (>400 pC N^−1^) as summarized in recent reviews,^12^, ^14^ we focus on the discussion on intrinsic bulk piezoelectric response in this progress report. Recent studies show that these polymers are ideal for flexible and biocompatible applications in energy harvesters, sensors, actuators, and so on.^14^, ^15^, ^16^, ^17^, ^18^, ^19^, ^20^, ^21^, ^22^, ^23^, ^24^, ^25^, ^26^, ^27^, ^28^, ^29^, ^30^, ^31^, ^32^, ^33^, ^34^ To foster these promising applications, it demands a large improvement of the modest piezoelectric coefficients of the polymers, which directly determines the efficiency of piezoelectric energy harvesting and the performance of sensors and actuators.^18^, ^20^, ^21^ However, despite decades of intensive research, there is a lack of molecular approaches to improve the intrinsic piezoelectric responses of ferroelectric polymers. Although the approaches by increasing the β phase (all‐*trans* conformation) fraction and/or crystallinity^35^, ^36^ have been popularly employed, some contrasting experimental results have been reported.^37^, ^38^, ^39^, ^40^, ^41^, ^42^, ^43^, ^44^, ^45^, ^46^, ^47^, ^48^ Moreover, the highest intrinsic piezoelectric coefficient *d*_33_ remains around −30 pC N^−1^ for ferroelectric polymers. It is believed that poor understanding of the origin of negative piezoelectric coefficient in PVDF‐based ferroelectric polymers significantly hampers the development of effective approaches to enhance their piezoelectricity in spite of growing interest for nearly 50 years.

Given that semicrystalline polymers typically consist of amorphous,^37^, ^38^ crystalline,^41^, ^45^, ^49^, ^50^ and amorphous–crystalline interfacial regions,^48^ three main microscopic models have been proposed to explain the piezoelectric behavior of PVDF‐based ferroelectric polymers. Surprisingly, all three models, despite controversy in nature, predict a *d*_33_ value of about −30 pC N^−1^ for P(VDF‐TrFE)s, which is consistent with the experimental data. Very recently, morphotropic phase boundary (MPB) has been discovered in P(VDF‐TrFE)s near the equiconcentration compositions (49 mol% ≤ VDF ≤ 55 mol%),^50^ leading to the state‐of‐the‐art *d*_33_ value of −63.5 pC N^−1^. The presence of MPB in the crystalline regions of the polymers is particularly interesting, which leads to the speculation of a purely crystalline origin of piezoelectricity recalling the semicrystalline nature of polymers discussed here.^51^, ^52^ In this progress report, we review these theoretical models and address the origin of negative longitudinal piezoelectric coefficient by the comparison between theoretical results and experimental data. We analyze the formation of MPB in ferroelectric polymers from molecular perspectives. It is anticipated that MPB is a general phenomenon for ferroelectric polymers with rich crystalline conformations, and molecular engineering of these conformations in the phase diagram may inspire future explorations of new MPB polymers with high piezoelectric responses. Our discussions are focused on bulk piezoelectric responses of polymers in this article.

## 2 Crystal Structures and Theoretical Models of Piezoelectricity in Ferroelectric Polymers

Semicrystalline polymer can be considered as a composite model, in which the nanoscale size crystallites are embedded in an amorphous matrix. One critical prerequisite to tune the piezoelectric response of these polymers is to understand precisely which domain plays a dominant role in determining piezoelectricity. There are basically three models available according to the literature: the dimensional model based on the amorphous region;^37^, ^38^ the electrostriction model based on the crystalline region;^41^, ^45^, ^49^, ^50^ and the modified electrostriction model including both crystalline and crystalline–amorphous interfacial coupling.^48^

### 2.1 Crystal Structure

**Figure 2:**
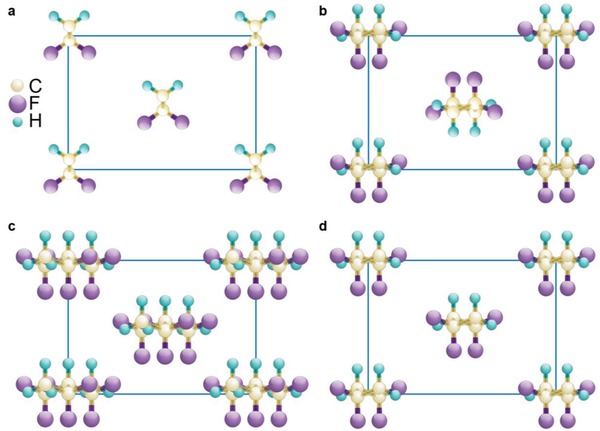
Crystal structure of PVDF. a) The β phase. b) The α phase. c) The γ phase. d) The δ phase.

It is known that PVDF can crystallize into at least five different crystalline phases depending strongly on the fabrication conditions.^53^, ^54^, ^55^, ^56^, ^57^, ^58^, ^59^, ^60^, ^61^, ^62^, ^63^, ^64^ Here, we show the four most studied phases in **Figure** **2**. Without any external treatment during crystallization, PVDF is in the α phase (Figure 2b, TGTG¯ conformation, T: *trans*, G: *gauche*), which is centrosymmetric and paraelectric‐like.^54^, ^59^ The well‐known ferroelectric phase—the β phase (Figure 2a, all *trans* conformation)—can be achieved by mechanical drawing, electrical poling, or defect modifications.^54^, ^56^, ^59^ It is believed that the highest ferroelectric and piezoelectric responses are related to this polar phase with the maximum dipole moment.^6^ The ferroelectric phase transition in PVDF cannot be observed as the Curie temperature is believed to be higher than the melting temperature. The discovery of ferroelectric phase transition in P(VDF‐TrFE) copolymer not only elucidates the ferroelectric nature of PVDF but also provides a mechanically free route to stabilize the ferroelectric β phase.^65^, ^66^, ^67^, ^68^, ^69^, ^70^, ^71^, ^72^, ^73^, ^74^, ^75^, ^76^, ^77^, ^78^, ^79^, ^80^, ^81^, ^82^, ^83^, ^84^ The effect of mechanical force on the piezoelectric response of ferroelectrics depends strongly the material of interest. Here, P(VDF‐TrFE) exhibits a slightly higher *d*_33_ value than PVDF at the expense of the reduction of the Curie temperature.^12^ In addition to the β phase, the ferroelectric characterization of the δ phase or a polar version of the α phase (Figure 2d) has been revisited^63^ and solid‐state processing of the δ phase PVDF was reported very recently.^64^ The δ phase was demonstrated in nanoscale films rather than bulk films.^63^ In the case of bulk samples fabricated by hot pressing, the product is a mixture of the α phase and the δ phase, in which *d*_33_ was calculated through the modified electrostriction model.^64^ The strong spontaneous polarization of PVDF and P(VDF‐TrFE) enables a wide range of applications in the low‐cost and flexible organic electronic devices such as organic solar cells,^85^, ^86^ nonvolatile ferroelectric memory,^87^, ^88^, ^89^, ^90^, ^91^, ^92^, ^93^ and field‐effect‐based devices.^86^, ^94^, ^95^

Polymer crystallography generally corresponds to the ideal limit condition disregarding concomitant structural defects (disorder) and complex morphology (e.g., a composite of amorphous, crystalline, and intermediate regions) existing in real polymers.^96^ Typically, the number of reflections available is limited and the Bragg peaks are very broad at high values of the 2θ diffraction angle, which significantly increase the difficulty of crystallographic analysis. As a result, it usually yields a high disagreement factor (≈10–30%) in structural refinements of ferroelectric polymers based on current diffraction techniques,^53^, ^57^, ^74^, ^77^, ^84^ which is in stark contrast to inorganic ceramics and crystals. To reach a better agreement with the experimental data, more complicated models were considered taking into account the tiltings or deflections of chains.^53^, ^74^, ^76^, ^77^, ^79^, ^85^, ^97^

Given that the physical properties of polymers depend on the arrangement of constituent monomers at the single chain scale, a more practical paradigm is to focus on the constituent monomers and microstructures instead of the crystallographic structures. Polymer chains in crystals usually adopt a preferred state with a specific sequence of bonds and torsion angles which corresponds to the low‐energy state.^98^ Chain conformations stem from different spatial arrangements of the atoms in a molecule of a given constitution, while the configuration may arise from rotation around single bonds.^6^ The conformation assumed by polymer molecules in the crystalline state depends on the configuration of the stereoisomer along the chains. The change in the conformation indicates a change in shape of a given molecule due to the torsion of single bond. That is one main reason why different crystalline conformations of PVDF exhibit distinct physical behaviors.^6^ Such interconversion of conformational isomerism may be in essence accompanied by symmetry breaking.

### 2.2 Dimensional Model

In the dimensional model, the dipoles are assumed to be fixed and the crystallites are supposed to be independent of the amorphous matrix.^37^, ^38^ In this regard, applying a uniaxial stress σ_3_ results in a reduction of film thickness and thus an enhancement of capacitance (Figure 1d) and a corresponding change in charges on the electrodes. As a result, there exists a built‐in field with its direction being the same as the polarization direction, which is in contrast to that observed in displacive ferroelectric perovskite such as PZT (Figure 1c).^48^

The piezoelectric effect in polymers can therefore be described in terms of the deformation of the amorphous regions. The original version of this model gives rise to a complex formula which depends on various parameters, leading to various measurements and unfortunately increasing the uncertainties.^37^ Later, the expression of this model was simplified and *d*_33_ can be estimated through^38^

(1) $$d_{33}=\frac{\partial P_{3}}{\partial \sigma_{3}}\approx -\frac{P_{\text{r}}}{Y}$$

where *P*_r_ is the remanent polarization and *Y* is the Young's modulus. According to Equation (1), the dimensional effect is much weaker in inorganic perovskites because perovskite ceramics are much stiffer (larger *Y*) than polymer materials. Consequently, this model can account for two‐third of the piezoelectric activity with the remaining contributions from the dipole moments of the crystalline regions.^37^

### 2.3 Electrostriction Model

When an electric field is applied onto a dielectric material, dimension changes due to the internal stress caused by the force of the electric field *E* on charges.^1^, ^27^ This effect is called electrostriction occurring in both crystalline and amorphous regions.^1^ Depending on materials, electrostrictive behavior can be hysteretic or anhysteretic with electric field. Relaxor ferroelectrics exhibit anhysteretic electrostriction, which is of importance to dimensional stability and reproducibility for high precision actuation using electrostrictics.^99^ In contrast to the piezoelectric effect (which has a linear dependence on the field), electrostriction scales with *E* ^2^ and does not depend on the field direction, and the strain *S*_3_ can be determined according to *S*_3_ = *Q*_33_ *P* ^2^, where *P* is the polarization. In ferroelectrics, the longitudinal piezoelectric coefficient *d*_33_ can be determined by the electrostriction biased by ferroelectricity^45^, ^50^

(2) $$d_{33}=2Q_{33}\varepsilon_{\text{r}}\varepsilon_{0}P_{\text{r}}$$

where ε_r_ and ε_0_ are the relative and vacuum permittivity, and *Q*_33_ is the electrostrictive coefficient. Recalling that the long‐range ferroelectric order in polymers arises only from the crystalline regions,^5^ the validity of Equation (2) in ferroelectric polymers disregards the amorphous regions, therefore contradicting with the dimensional model.^37^, ^38^

The electrostriction in PVDF was firstly studied at the beginning of 1970.^100^, ^101^, ^102^ The first use of electrostriction concept to explain the piezoelectricity was proposed in 1975 (see details about the relation between electrostriction and piezoelectricity in Section 3.3), in which the contributions from the amorphous and crystalline regions were not discussed.^44^ The electrostriction model proposed by Furukawa and Seo exhibits a good agreement between experimental data and theoretical prediction by using Equation (2).^45^ However, they attributed electrostriction in PVDF and P(VDF‐TrFE) to the dimensional effect arising from the amorphous regions.^45^ In addition, the measurement of *Q*_33_ was carried out in the ferroelectric phase of polymers,^45^, ^48^ in which the extrinsic contributions from ferroelectric switching and domain wall motion^1^ were not taken into account.

### 2.4 Modified Electrostriction Model

**Figure 3:**
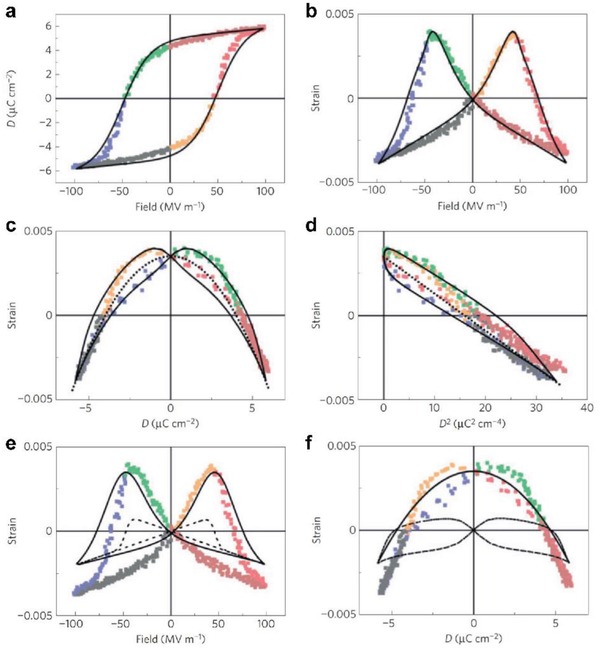
a) Ferroelectric displacement *D* as a function of applied electric field *E* (1 Hz, triangular waveform). The solid curve is a fit according to the model in ref. 48. Strain as a function of b) field (*S*_3_–*E*), c) displacement (*S*_3_–*D*), and d) displacement squared (*S*_3_–*D* ^2^). Different types of colors correspond to different measurement steps. The dotted lines are the fits according to *S*_3_ = *Q*_33_ *D* ^2^. The black solid lines are a fit according to Equation (3). e,f) The experimental strain and the contributions of the electrostrictive term, *Q*_33_ *D* ^2^ (solid lines), and the coupling term (dotted lines), as a function of electric field and electric displacement. The experimentally observed hysteresis at high fields was attributed to the additional term in Equation (3).^48^ Adapted with permission.^48^ Copyright 2015, Nature Publishing Group.

Since Equation (2) accounts for the piezoelectricity with a crystalline origin, the understanding of the electric‐field‐induced structural change in the crystalline regions is highly desired in order to provide further evidence. In this regard, the recent work by Katsouras et al. is of particularly importance as they developed an in situ X‐ray measurement on the strain response as a function of electric field and real time.^48^ As a result, the electric‐field‐driven strain can be measured according to the lattice change determined by X‐ray measurements (**Figure** **3**). Very interestingly, the strain response in P(VDF‐TrFE) 65/35 mol% copolymer displays a butterfly feature (Figure 3b), characteristic of ferroelectric instability. Moreover, not only the shape but also the strain value (Figure 3b) bear a surprising resemblance to those reported by Furukawa and Seo who measured macroscopically the change in film thickness with the application of an electric field.^45^ This experimental evidence thus strongly supports that the crystalline regions may be mainly responsible for the electromechanical response of PVDF and P(VDF‐TrFE).

The agreement between the macroscopic and microscopic strain results implies that Equation (2) may explain the piezoelectricity occurring in the crystalline domain of polymers. However, Katsouras et al. found that it is unable to fully account for the observed strain data (Figure 3c,d) by using Equation (2). They found a hysteretic behavior when they measured *Q*_33_ (Figure 3c); they also found that electrostrictive strain from the crystalline regions at high electric fields is smaller than the total strain value (Figure 3e,f). As a result, they modified Equation (2) taking into account the electromechanical contribution from the crystalline–amorphous interfacial regions^48^ on the basis of the electrostrictive strain in the crystalline regions. This is so‐called modified electrostriction model in which *d*_33_ is expressed as

(3) $$d_{33}=2Q_{33}\varepsilon_{\text{r}}\varepsilon_{0}P_{\text{r}}+d_{\text{coupling}}$$

where *d*_coupling_ is the additional contribution arising from the crystalline–amorphous interfacial coupling. As a result, it is found that Equation (3) can be used to describe the experimentally observed behavior (Figure 3c–f). Compared to the results from Furukawa and Seo, the main differences on P(VDF‐TrFE) 65/35 mol% copolymers arise from the data on *Q*_33_ and *P*_r_. Specifically, *Q*_33_ measured by the macroscopic approach is −2.1 m^4^ C^−2^ 45 while it is −1.5 m^4^ C^−2^ collected by the microscopic method.^48^ *P*_r_ is about 0.086 C m^−2^ in bulk film (i.e., 10–30 µm)^45^ versus ≈0.043 C m^−2^ measured in nanosize film (i.e., 450 nm).^48^ ε_r_ varies little from 10.4 in ref. 45 to 10.0 in ref. 48. Except the sample difference in terms of fabrication condition and film thickness, the extrinsic contributions from ferroelectric switching and domain wall motion^102^ were not addressed in the electrostrictive measurements. Moreover, it was reported that the coupling term in Equation (3) can be a major contribution accounting for two‐third of *d*_33_ while the intrinsic electrostriction only constitutes one‐third of the total piezoelectric response.^48^

**Figure 4:**
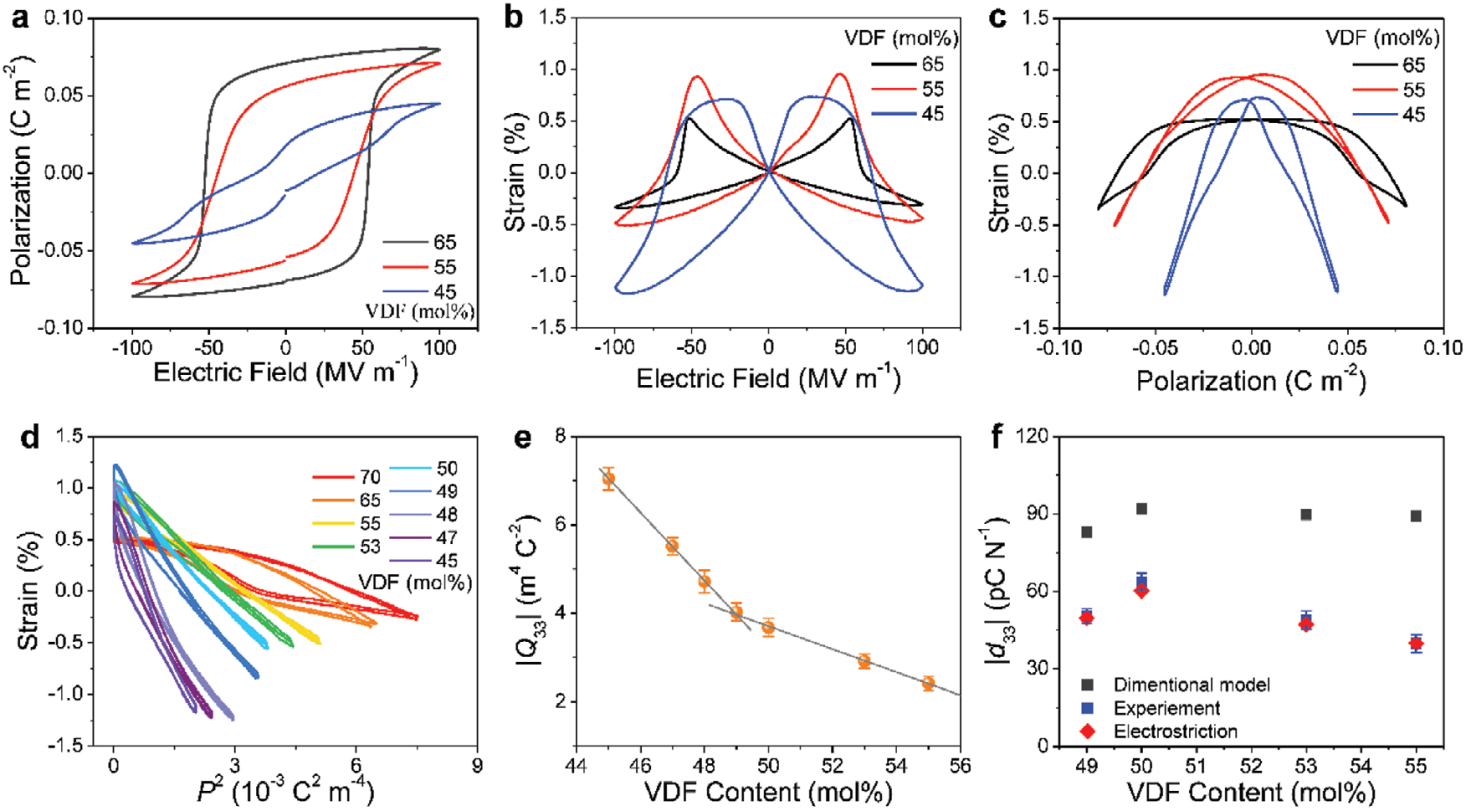
a) Polarization against electric field hysteresis loops measured by using a triangular ac electric field of 1 Hz at room temperature. b) Simultaneous strain–electric field response. c) *S*_3_–*P*. d) *S*_3_–*P* ^2^. Typical P(VDF‐TrFE) compositions are selected in (a)–(d) to provide a better view. e) *Q*_33_ as a function of VDF content. The lines are guide for the eyes. f) Comparison between *d*_33_ measured at room temperature and theoretical predictions from the dimensional model and electrostriction model. Adapted with permission.^50^ Copyright 2018, Nature Publishing Group.

## 3 Origin and Tuning of Negative Longitudinal Piezoelectric Coefficient in P(VDF‐TrFE)

In this section, we address the importance of electrostrictive measurements and compare the results between electrostriction model and piezoelectric data observed in P(VDF‐TrFE) copolymers (**Table** **1**). We discuss the role of electrostrictive coefficient in determining the contributions from the crystalline regions of the polymers and review the high‐temperature electrostrictive measurement in piezoelectric polymers. A rather flat energy landscape between energetically degenerate *trans*‐planar (all *trans* conformation) and 3/1‐helical phases [(TG)_3_ or (TG¯ )_3_ conformation] is achieved near the MPB, where the enhanced piezoelectric response was observed in P(VDF‐TrFE). We also discuss the possibilities of formation of MPB through the single chain model or mixed chains model. We anticipate that MPB may be a general phenomenon in polymer materials, which may attract more attention to develop high‐performance piezoelectric polymers.

**Table 1:** Comparison of PVDF‐based polymers with other typical piezoelectric polymers

Material	Thickness [µm]	*d*_33_ [pC N^−1^]	Ref.
PVDF‐based polymers			
Drawn PVDF	10–30	−26.0	45
Spin‐coated PVDF	0.45	−37.7	48
Undrawn P(VDF‐TrFE) 81/19 mol%	19–22	−18.0	103
Drawn P(VDF‐TrFE) 75/25 mol%	20–80	−38.0	35
Spin‐coated P(VDF‐TrFE) 75/25 mol%	0.05	−21.9	104
LB^a)^ P(VDF‐TrFE) 70/30 mol%	0.015	−22.0	105
Ultrathin P(VDF‐TrFE) 70/30 mol%	0.004	−46.4	106
Drawn P(VDF‐TrFE) 65/35 mol%	10–30	−35.0	45
Undrawn P(VDF‐TrFE) 65/35 mol%	10–30	−30.0	45
Spin‐coated P(VDF‐TrFE) 65/35 mol%	0.45	−31.4	48
Drawn P(VDF‐TrFE) 52/48 mol%	–	−28.0	107
Undrawn P(VDF‐TrFE) 52/48 mol%	10–30	−44.0	45
Undrawn P(VDF‐TrFE) 52/48 mol%	20	−30.0	108
Undrawn P(VDF‐TrFE) 50/50 mol%	60	**−63.5**	50
Other semicrystalline polymers			
Nylon 11	30–35	−3.9	109
Nylon 13	30–35	−4.1	109
Parylene‐C	50	−2.0	110
Polyurea	0.5	10.0 (*d*_31_ ^b)^)	111
Amorphous piezoelectric polymers			
Polyimide (β‐CN)APB/ODPA^c)^	30	−16.5	112
Polyimide (β‐CN)APB/ODPA	–	−2.7	113
Polyvinyl chloride (PVC)	1000	−1.0	114
P(VDCN/VAc)^d)^	–	5.0 (*d*_31_ ^b)^)	115
P(AN‐MA)^e)^	10–15	3.0 (*d*_31_ ^b)^)	116
Poly(meth)acrylate	–	1.5 (*d*_31_ ^b)^)	117
Poly(1‐bicyclobutanecarbonitrile)	25	0.3 (*d*_31_ ^b)^)	118

^a)^ LB stands for Langmuir–Blodgett

^b)^ *d*_31_ is the transverse piezoelectric coefficient. *d*_31_ was listed here because no *d*_33_ data were reported in these polymers

^c)^ Polyimide (β‐CN)APB/ODPA: prepared from 2,6‐bis(3‐aminophenoxy) benzonitrile ((β‐CN)APB) and 4,4′ oxidiphthalic anhydride (ODPA)

^d)^ P(VDCN/Vac): poly(vinylidene cyanide‐*alt*‐vinyl acetate)

^e)^ P(AN‐MA): poly(acrylonitrile‐*co*‐methyl acrylate).

### 3.1 The Role of Electrostrictive Coefficient

According to Equations (1)–(3), the electrostrictive coefficient *Q*_33_ is particularly important as all the other parameters such as ε_r_, ε_0_, and *P*_r_ can be measured directly. (**Figures** 3a–d,**4**a–d summarize the general approach to obtain *Q*_33_ according to previous works.^45^, ^48^ It usually requires the measurement of polarization (Figures 3a,4a) and strain (Figures 3b,4b) simultaneously. Considering these curve evolution with the decrease of VDF content, polarization hysteresis loops evolve from ferroelectric to antiferroelectric‐like type (Figure 4a), which is accompanied by a remarkable smearing of the butterfly peaks (i.e., VDF = 45 mol%, Figure 4b). The copolymers with antiferroelectric‐like loops (VDF < 49 mol%) are not in a true antiferroelectric phase^119^, ^120^, ^121^, ^122^, ^123^ due to the absence of typical strain response characteristic of antiferroelectric materials (Figure 4b). In addition, Figure 4b shows that the emergence of relaxor behavior for VDF ≤ 55 mol% largely improves the strain response, as relaxor ferroelectrics are known to exhibit remarkably larger electrostriction strain than normal ferroelectrics.^99^, ^124^, ^125^, ^126^, ^127^

The electrostriction can be shown in strain–polarization (*S*_3_–*P*) curves (Figure 4c), which are plotted by eliminating the electric field in Figure 4a,b.^45^, ^48^ The key finding in *S*_3_–*P* response of P(VDF‐TrFE) copolymer is that the *S*_3_–*P* curve is completely hysteretic for VDF = 65 mol% and higher VDF concentrations regardless of high or low electric fields. Figure 4c shows that such irreversible parts at high fields can evolve into reversible parts as long as the relaxor behavior is induced in P(VDF‐TrFE) copolymer (VDF ≤ 55 mol%). The hysteretic behavior observed in P(VDF‐TrFE) 65/35 mol% was attributed to the interfacial strain coupling, according to the previous work.^48^ Indeed, the irreversible parts may arise from the extrinsic contributions from domain wall motions and domain switching,^128^ which can lead to large uncertainties in the deduced *Q*_33_ value. By contrast, the reversible parts at high fields (VDF ≤ 55 mol%) may be related to relaxor behavior observed in these compositions as relaxors are usually known to exhibit reversible electrostrictive responses at high fields.^99^, ^124^, ^125^, ^126^, ^127^

To further show the effect of hysteretic behavior on affecting *Q*_33_, *S*_3_–*P* ^2^ curves are plotted in Figure 4d, where the slope of the curve corresponds to *Q*_33_. The copolymers with typical ferroelectric compositions (i.e., VDF = 65 and 70 mol%) can have two distinct *Q*_33_ values based on a significant hysteresis at high electric fields (i.e., 100 MV m^−1^). In this regard, the extracted *Q*_33_ would be in a range from −1.29 ± 0.16 to −2.28 ± 0.04 m^4^ C^−2^ for VDF = 65 mol%, which is obviously not the case. Such hysteretic effects are nearly eliminated for VDF ≤ 55 mol%, where relaxor behavior appears (VDF ≤ 55 mol%). The compositional evolution from relaxor to normal ferroelectric as a function of VDF composition is accompanied by a large hysteresis increase in the electrostrictive response. Similar results were already reported in various piezoelectric ceramics such as Pb(Mg_1/3_Nb_2/3_)O_3_‐PbTiO_3_,^99^ (Bi_0.5_Na_0.5_)TiO_3_‐BaTiO_3_‐(Sr_0.7_Bi_0.2_)TiO_3_,^129^ and NaNbO_3_‐BaTiO_3_.^130^ As VDF content decreases, the slope of *S*_3_–*P* ^2^ curves increases substantially (Figure 4d), where a slope change at VDF = 49 mol% (Figure 4e) is attributed to the disappearance of ferroelectric instability.^50^ To reduce or eliminate the hysteretic effect, the measurement of *Q*_33_ above the Curie temperature is highly desired (see Section 3.2).

Having *Q*_33_, the comparison between the experimental piezoelectric data and theoretical models (Figure 4f) can be made. Interestingly, it is found that Equation (2) is satisfied for the MPB compositions (49 mol% ≤ VDF ≤ 55 mol%), which indicates that the electrostriction model can account for negative longitudinal piezoelectric coefficient in P(VDF‐TrFE)s. This result demonstrates that the crystalline–amorphous coupling previously thought to be a major contribution to piezoelectricity^48^ may play a minor role in driving the negative longitudinal piezoelectric coefficient. On the other hand, the calculated *d*_33_ based on the dimensional effect are significantly larger than the experimental data. Such substantial deviations therefore discard the interpretation of negative longitudinal piezoelectric coefficient in terms of the deformation accommodated by the amorphous domain of P(VDF‐TrFE)s.^37^, ^38^ In addition, the Maxwell‐stress‐induced strain^131^ was estimated to be more than one order of magnitude weaker than the electrostrictive strain of ≈0.003–0.010 at the same field, which also plays a negligible role.

### 3.2 High‐Temperature Electrostrictive Measurements

The determination of intrinsic electrostrictive coefficients of ferroelectrics usually requires the measurement to be done at a high electric field and a high temperature (above the Curie temperature) to avoid the extrinsic contributions from ferroelectric switching and domain wall motion.^102^ PVDF‐based ferroelectric polymers usually show strong degradation of their physical properties at high temperatures (around 100 °C), where the segments and even molecular chains may be free to rotate or move. Therefore, the high‐temperature electrostrictive measurements on ferroelectric polymers are technically challenging, which are limited by a lossy paraelectric phase with a significantly reduced breakdown field. In a lossy paraelectric phase, the measured polarization is not intrinsic due to the extrinsic contributions from the dramatic increase of electrical conductivity, which leads to large uncertainties in extracting *Q*_33_, even though the strain response can be obtained.

**Figure 5:**
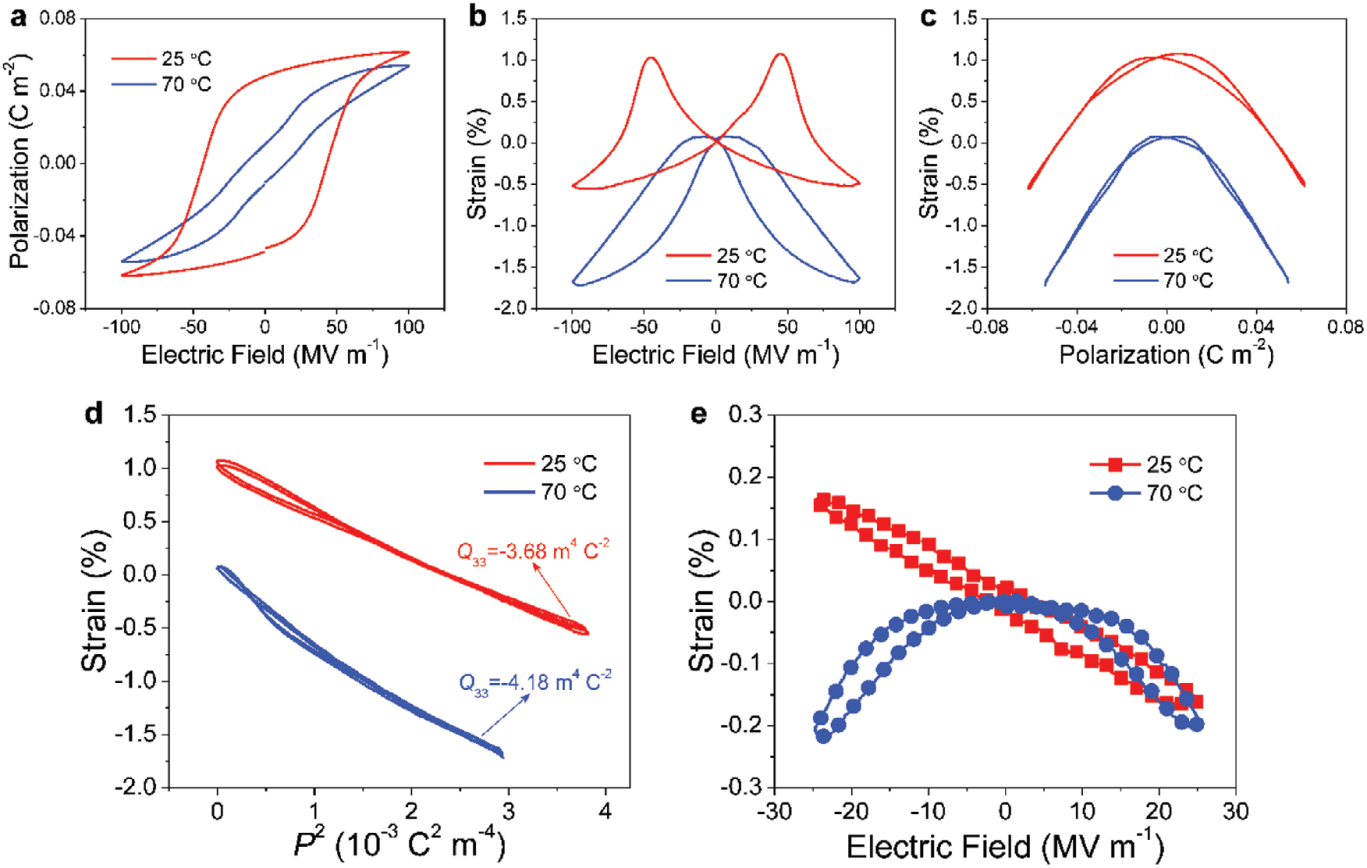
a) Polarization versus electric field loops collected at 70 °C. b) Electric‐field‐induced strain *S*_3_ at 70 °C. c) *S*_3_–*P*. d) *S*_3_–*P* ^2^. e) Electric‐field‐induced strain at small fields at 70 °C. All the data are obtained from P(VDF‐TrFE) 50/50 mol% copolymer. Adapted with permission.^50^ Copyright 2018, Nature Publishing Group.

The high‐temperature electrostrictive data are very useful to justify the room‐temperature results. **Figure** **5** summarizes the results in P(VDF‐TrFE) copolymers with a typical MPB composition of 50/50 mol%, which exhibits the largest piezoelectric response (|*d*_33_| = 63.5 pC N^−1^)^50^ nearly doubling the previous results (Table 1). At the paraelectric phase (70 °C, just above the Curie temperature of ≈65 °C), Figure 5a shows a slim polarization loop, indicative of high‐quality polymer films with nearly no conductive loss. We note that the loop can evolve into a much slimmer shape as long as the frequency is increased. The increase in the frequency may also lower the contribution from ionic conduction in polymer materials. Figure 5b shows that the strain response evolves from a typical butterfly at the ferroelectric phase (25 °C) into a shape characterized by remarkably flatter butterfly peaks at the paraelectric phase (70 °C), which is attributed to order–disorder phase transition. As expected, the hysteresis in *S*_3_–*P* curve at high temperatures is reduced considerably compared to that at room temperature (Figure 5c). Moreover, it is found that the slope of *S*_3_–*P* ^2^ curve measured at the paraelectric phase changes only slightly with respect to that deduced at the ferroelectric phase (Figure 5d). Specifically, *Q*_33_ extracted at 70 °C is −4.18 ± 0.32 m^4^ C^−2^ while it is −3.68 ± 0.21 m^4^ C^−2^ at 25 °C. Consequently, the calculated *d*_33_ is −68.3 pC N^−1^ using a *Q*_33_ value of −4.18 m^4^ C^−2^, which is nearly the same as the experimental result of −63.5 pC N^−1^.^50^ The copolymer at small fields (i.e., 25 MV m^−1^) and at 70 °C only displays the typical quadratic behavior which is characteristic of the electrostriction. We note that the field of 25 MV m^−1^ is well below the coercive field of about 50 MV m^−1^, which is therefore frequently used to study strain responses at small fields of ferroelectric polymers. The magnitude of the electrostrictive strain (70 °C) is slightly higher than the piezoelectric strain measured at 25 °C (Figure 5e). The paraelectric phase therefore shows no piezoelectric activity, which is contrary to previous results showing that robust piezoelectricity still exists just above the Curie temperature.^43^, ^69^ The high‐temperature electrostrictive results unambiguously show the negative sign of electrostrictive effect and support that Equation (2) can fully account for the longitudinal piezoelectric coefficients in P(VDF‐TrFE)s.

### 3.3 The Relation between Electrostriction and Piezoelectric Effect

Assuming that applied stresses, temperature, etc., are constant, the strain–field relation under a single domain structure can be generally written as

(4) $$S_{3}=d_{33}E+M_{33}E^{2}+\text{higher}\;\text{orders}$$

where *M*_33_ is the electrostrictive coefficient. Here, we only consider the first and second terms and their role in affecting the total strain response of materials. At small fields, the linear term in Equation (4) arising from the converse piezoelectric effect plays the major contribution while the quadric term due to the electrostrictive effect is negligible. At high fields, such strain contribution is reversed with the electrostrictive effect being dominant.

In the real case, *d*_33_ and *M*_33_ are not constant, both of which strongly depend on the electric field. There are always other contributions to the total strain response, i.e., domain wall motion. In the case of typical strain measurement under bipolar fields (above the coercive field), the domain wall motion related to the ferroelectric domain switching cannot be avoided in the ferroelectric phase.^128^ This is one main reason why measuring intrinsic electrostrictive coefficients always requires high field and particularly high temperature above the Curie temperature. In ferroelectric phase, the chains cannot easily be rotated without high poling field due to the existence of large‐scale ferroelectric domains. This may lead to the depression of electrostrictive response inside the crystalline lattice especially at small fields. On the contrary, the polymer chains become mobile and ready to rotate in the paraelectric phase which only exhibits only electrostriction with no piezoelectric activity.

Previous conclusion that the negative piezoelectric strain either completely^45^ or mainly^48^ results from electrostrictive type depending on the agreement factor between the experimental data and Equation (3), as analyzed above. To avoid potential confusion or misunderstanding from Equation (4), it is suggested to make a comparison between the strain data at small fields and at high temperatures above the Curie temperature (which is purely electrostrictive) and the one at small fields and at low temperature (which is mostly piezoelectric) in order to clarify the relation between piezoelectric and electrostrictive effects in ferroelectrics. Interestingly, Figure 5e shows that the temperature‐triggered strain evolution from nearly linear piezoelectric response in the ferroelectric phase to a parabola electrostrictive type in the paraelectric phase. This behavior is reminiscent of the results found in copolymer/terpolymer blend, where tuning of copolymer fraction can lead to a similar strain change from piezoelectric to electrostrictive response (see Section 3.5). Moreover, Figure 5e shows that the electrostrictive strain at 25 MV m^−1^ in the paraelectric phase can fully account the piezoelectric strain under the same field in the ferroelectric phase. These results strongly support that the piezoelectric strain may nominally arise from electrostrictive type in P(VDF‐TrFE). When P(VDF‐TrFE) copolymer is cooled from paraelectric phase, the phase transition starts when the strain curve with a parabola shape becomes asymmetric; that is, the strain sign tends to change when a negative field is on. P(VDF‐TrFE) is completely in ferroelectric phase when the strain curve is linear showing only piezoelectric response. In particular, previous ab initio calculations have clearly shown a negative sign of electrostrictive effect, which was attributed to the rearrangement of atomic nuclei in response to external electric field.^47^ The computation results also showed that Equation (2) can be satisfied. Previous phenomenological claims that electrostriction is responsible for piezoelectricity which were mainly deduced on the basis of Equation (3).^45^, ^48^, ^50^ This does not necessarily mean that piezoelectricity and electrostriction can convert into each other as they belong to first and second‐order effects as shown in Equation (4). Indeed, either the validity of Equation (3) or temperature‐dependent strain data at small fields can nominally account for the sign and magnitude of piezoelectricity in PVDF‐based ferroelectric polymers. Therefore, previous works^45^, ^48^, ^50^ may mostly act as the phenomenological developments. At current research stage, no definitive answers to the microscopic picture have been revealed. We therefore hope our progress report can inspire further studies on the basis of previous works.

### 3.4 Tuning the Piezoelectric Response in P(VDF‐TrFE) by MPB Approach

MPB, a boundary separating two competing ferroelectric phases in the phase diagram, is one of the most vital concepts in ferroelectric materials.^51^, ^52^, ^132^, ^133^, ^134^, ^135^, ^136^, ^137^, ^138^, ^139^ Exciting physics including the colossal physical responses to external stimuli have been revealed at such phase boundaries. The most technologically useful piezoelectric materials are all designed based upon MPB, which are pivotal to modern smart technologies that integrate ultrasensitive sensing with high‐precision actuation functions in biomedical devices, telecommunications, and electronics. MPB was discovered in PZT more than half a century ago^140^ and so far only occurs in a few ceramic piezoelectric materials such as PZT, Pb(Zn_1/3_ Nb_2/3_)O_3_‐PbTiO_3_ (PZN‐PT), and Pb(Mg_1/3_Nb_2/3_)O_3_‐PbTiO_3_ (PMN‐PT), each of which has stimulated unprecedented interest in both fundamental research and practical applications. Surprisingly, this physical concept has never been realized in organic materials until the very recent discovery.^50^

Liu et al. have demonstrated the chain tacticity‐induced MPB in the ferroelectric P(VDF‐TrFE) copolymers, which provides a unique molecular approach to MPB.^50^ They have unambiguously confirmed the presence of the MPB in the ferroelectric polymers via comprehensive characterization in conjunction with the first‐principles calculations. For the first time, MPB has been analyzed at the molecular level. In addition, a record performance has been achieved, i.e., a longitudinal piezoelectric coefficient of −63.5 pC N^−1^ from the copolymer with the morphotropic composition, which nearly doubles the state‐of‐the‐art results on piezoelectric polymers (Table 1). This work offers a molecular engineering approach to improve the intrinsic piezoelectric properties of ferroelectric polymers.

Design of a MPB requires either two constituent phases that coexist and compete with each other or an intermediate low‐symmetry phase to form a transition region, which intimately bridges two different phases on both sides. Accordingly, a mixture of two different phases that simply coexist without any competition with each other in a piezoelectric system may not necessarily lead to the formation of MPB. That is probably the main reason why there are only few MPB piezoelectric materials. As MPB allows the interconversion of nearly energetically degenerate phases, applying an appropriate electric field tunes the constituent phase fractions, and consequently, giving rise to large piezoelectric responses.^141^, ^142^ Several examples of MPBs are listed below, which are characterized by the intimate coexistence of two competing phases: Sm‐doped BiFeO_3_,^143^ (1−*x*)BiTi_3/8_Fe_2/8_Mg_3/8_O_3_‐*x*CaTiO_3_,^144^ (1−*x*)BiFeO_3_‐*x*PbTiO_3_,^145^ and so on.

**Figure 6:**
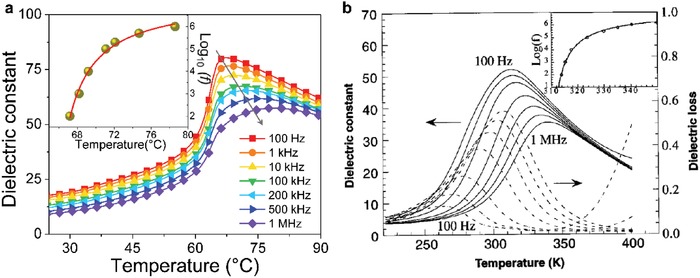
a) Temperature dependence of the dielectric constant of P(VDF‐TrFE) (VDF = 50 mol%), showing relaxor behavior.^50^ The gray arrow shows the dependence of the dielectric constant on the frequency *f* of the a.c. electric field upon heating. The inset shows a fit of the measured dielectric constant (dark yellow circles) with the Vogel–Folcher law (red solid line) such that ln*f* = ln*f*_0_−*E*_a_/*k*_B_(*T*_max_−*T*_f_), where *f* is the frequency, *f*_0_ is the attempt frequency, *E*_a_ is the activation energy, *k*_B_ is the Boltzmann constant, *T*_max_ is the dielectric peak temperature, and *T*_f_ is the freezing temperature. Adapted with permission.^50^ Copyright 2018, Nature Publishing Group. b) The dielectric constant (solid lines) and dielectric loss (dashed lines) as a function of temperature for P(VDF‐TrFE) 50/50 copolymer after irradiation at 120 °C.^125^ The frequency is (from top to bottom curves for dielectric constant and from bottom to top curves for dielectric loss): 100 Hz, 1 kHz, 10 kHz, 100 kHz, 300 kHz, 600 kHz, and 1 MHz. The inset shows the fitting of the Vogel–Folcher law, where the solid line is the fit and the circles are the data [the horizontal axis in the inset is temperature (in kelvin), and *f* is the frequency]. Adapted with permission.^125^ Copyright 1998, American Association for the Advancement of Science.

One of the critical reasons to discover the MPB in P(VDF‐TrFE)s is the finding of its relaxor behavior (**Figure** **6**a). Although P(VDF‐TrFE)s have been extensively investigated for nearly 40 years, their complete phase diagram has not been yet established, especially around VDF = 50 mol%. Numerous textbooks and reviews suggest that ferroelectric instability of P(VDF‐TrFE)s disappears in the proximity of VDF = 50 mol%.^83^, ^123^, ^146^ Unfortunately, little is known about why ferroelectric distortion becomes physically unstable, given that there are only very few studies on TrFE‐rich P(VDF‐TrFE)s. Moreover, previous reports differ significantly on the structural understanding of P(VDF‐TrFE)s in the TrFE‐rich region, which ranges from antiferroelectric‐like phase,^118^, ^119^, ^120^, ^121^, ^122^ “cooled” phase (tilting of long *trans* segments)^76^ to a mixed ferroelectric and non‐ferroelectric phase.^83^ The disappearance of ferroelectric instability is indicative of the abrupt crystal structure changes occurring near VDF = 50 mol%. Such behavior induced by changing the VDF compositions bears a resemblance to MPB, across which the crystal structure is known to change abruptly. Moreover, the discovery of relaxor behavior (VDF ≤ 55 mol%) in P(VDF‐TrFE)s offers further confidence of the existence of MPB in ferroelectric polymers. Actually, these findings are in line with well‐established practices in the field of inorganic MPB, e.g., the benchmark MPB piezoelectric materials PZN‐PT^132^, ^147^, ^148^ and PMN‐PT^149^, ^150^ in which the evolution from normal ferroelectric to relaxor with the decrease of the PT concentrations leads to the formation of MPB. Importantly, the nature of TrFE‐rich P(VDF‐TrFE) is resolved as relaxor ferroelectric, which has not been demonstrated previously.^69^, ^71^, ^123^ It is known that relaxors usually enable striking properties (i.e., ultrahigh piezoelectric coefficient^132^ and large dielectric responses^147^) and have peculiarly microscopic structures that are different from those of normal ferroelectrics. Moreover, it is found that relaxor property is intrinsic to P(VDF‐TrFE)s rather than extrinsically driven by defect modifications such as electron irradiation (Figure 6b) and introduction of bulky monomers.^125^, ^126^, ^127^, ^151^ As a result, the dielectric constant measured at room temperature shows the maximum (≈18) in P(VDF‐TrFE) 50/50 mol%,^50^ which provides evidence of MPB formation in P(VDF‐TrFE) copolymers because the presence of MPB improves dielectric responses of ferroelectrics. Moreover, P(VDF‐TrFE) 50/50 mol% also exhibits the largest dielectric response (≈76 at 1 kHz) near the Curie temperature of 69 °C.^152^ In addition, the detailed analysis on compositional dependence of dielectric response clearly shows the existence of phase boundary behavior.^152^

**Figure 7:**
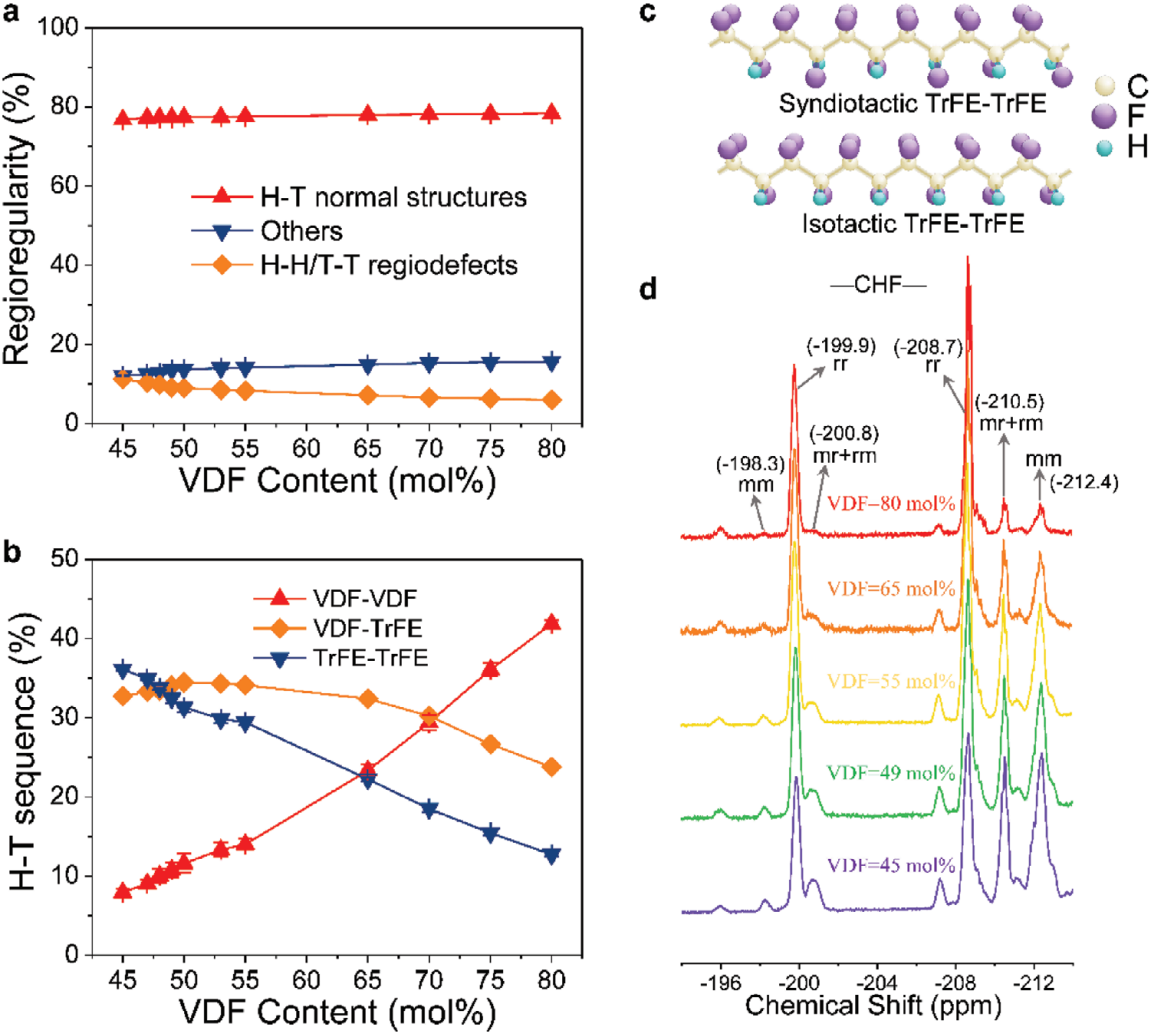
a) Unconditional probabilities of different regiosequences as a function of VDF content. b) Unconditional probabilities of normal *H*–*T* sequences consisting of the VDF‐VDF, VDF‐TrFE, and TrFE‐TrFE segments. c) Sketch of chain tacticity in TrFE‐TrFE segments. d) The —CHF— resonances in P(VDF‐TrFE) copolymers. The stereosequences were calculated by measuring the ratios of the integral intensities of the respective triad peaks in the —CHF— resonance region of ^19^F NMR spectra: For VDF‐TrFE segment, the syndiotactic (rr), heterotactic (mr+rm), and isotactic (mm) triad peaks center at −199.9, −200.8, and −198.3 ppm, respectively; For TrFE‐TrFE segment, the syndiotactic (rr), heterotactic (mr+rm), and isotactic (mm) triad peaks center at −208.7, −210.5, and −212.4 ppm, respectively. Adapted with permission.^50^ Copyright 2018, Nature Publishing Group.

Another important development in the microstructure shows that it is the change in chain tacticity evolution that leads to stabilization of a 3/1‐helical phase in P(VDF‐TrFE)s. Although chain tacticity was analyzed in other fluoropolymers,^54^, ^153^, ^154^, ^155^ the counterpart in P(VDF‐TrFE)s by nuclear magnetic resonance (NMR) studies^156^, ^157^, ^158^ was poorly defined. Random copolymerization of VDF and TrFE monomers may lead to the appearance of H‐H/T‐T (H, head; T, tail) regiodefects and regioirregular defects in P(VDF‐TrFE)s which can be determined from ^19^F NMR spectra. A long‐standing misunderstanding deduced from the first NMR work is that P(VDF‐TrFE) copolymers are predominant by H‐T VDF‐TrFE sequence for the VDF content ranging from ≈30 to 75 mol%.^156^ The poor NMR resolution (56.5 MHz) results in the absence of many resonance peaks (i.e., above 130 ppm). Moreover, only the —CF_2_— resonance area was considered disregarding the contributions from —CHF— resonance area.^156^ Consequently, the regioregularity was not properly described while the chain tacticity was not analyzed. This is also one of main reasons why theoretical works considered only VDF‐TrFE as the model of P(VDF‐TrFE) copolymers.^159^, ^160^, ^161^, ^162^ The regiosequences were explicitly determined based on the assignments of ^19^F NMR signals of P(VDF‐TrFE) copolymers (**Table** **2**). It can be seen that normal H‐T sequences consist of the VDF‐VDF, VDF‐TrFE, and TrFE‐TrFE segments (**Figure** **7**a). As the VDF content decreases, the TrFE‐TrFE units grow significantly, being even larger than the VDF‐TrFE counterparts (that remain nearly constant for VDF ranging from 45 to 65 mol%) for VDF < 49 mol%. This is accompanied by a remarkable decrease in the VDF‐VDF units (Figure 7b). These results clearly indicate that polymer chain becomes more PTrFE‐like [PTrFE: poly(trifluoroethylene)] for the copolymers with TrFE‐rich compositions, which is in contrast to the previous result.^156^ Figure 7d shows that the stereosequences are evaluated by analyzing the characteristic peaks of isotactic (mm), syndiotactic (rr), and heterotactic (mr+rm) triads.^50^, ^155^ It can be clearly seen in Figure 7d that the peak of isotactic (mm) triads in TrFE‐TrFE segments grows significantly as the VDF content decreases. This result indicates that the most favorable sequence for the TrFE‐TrFE segment is changed from syndiotactic to isotactic (Figure 7c) with decreasing the VDF content, which is in good agreement with the first‐principles calculations.^50^

**Table 2:** Assignments of ^19^F NMR signals for P(VDF‐TrFE)

Regioregularity	5C sequence	Designation	Chemical shift [ppm]
H‐T or T‐H	CF2CH2**CF2**CH2CF2	VDF‐VDF, H‐T	−93.2
	CF2CH2**CF2**CHFCF2	VDF‐TrFE, H‐T	−107.8
	CF2CHF**CF2**CHFCF2	TrFE‐TrFE, H‐T	−119.5 to −124.8
	CHFCF2**CHF**CF2CHF	TrFE‐TrFE, T‐H	−207.3 to −213.5
	CH2CF2**CHF**CF2CH2	TrFE‐VDF, T‐H	−197.5 to −201.5
H‐H/T‐T or T‐T/H‐H	CH2CH2**CF2**CF2CH2	VDF‐VDF‐VDF, T‐T/H‐H	−117.2
	CHFCHF**CF2**CF2CHF	TrFE‐TrFE‐TrFE, T‐T/H‐H	−124.8 to −130.0
	CH2CHF**CF2**CF2CH2	VDF‐TrFE‐VDF, T‐T/H‐H	−131.1
	CF2CF2**CHF**CHFCF2	VDF‐TrFE‐TrFE, H‐H/T‐T	−218.5 to −220.4
Others	CHFCH2**CF2**CH2CF2	TrFE‐VDF‐VDF, T‐T/ H‐T	−94.8 to −95.8
	CH2CH2**CF2**CH2CF2	VDF‐VDF‐VDF, T‐T/ H‐T	−96.3 to −97.8
	CF2CH2**CF2**CF2CHF	VDF‐VDF‐TrFE, H‐T/H‐H	−114.2

The monomers indicated by the ^19^F NMR signals are underlined. H‐H, head to head; H‐T, head to tail; T‐T, tail to tail.

### 3.5 MPB from Single Polymer Chains

**Figure 8:**
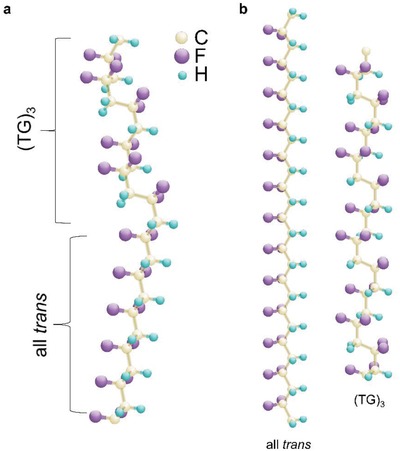
A ball‐and‐stick model corresponding to MPB formation: a) intramolecularly within a single molecular chain, and b) intermolecularly between mixed chains with all‐*trans* and 3/1 helix conformations. For simplicity, the model is illustrated using the structure of PVDF.

In this section, we mainly discuss about whether the boundary may form within a single molecular chain (**Figure** **8**a) or intermolecularly between polymer chains with distinct conformations (Figure 8b). Polymer blend may act as a very useful platform to testify this idea. To provide the similar evolution from normal ferroelectric to relaxor, it is rational to use normal ferroelectric copolymer [P(VDF‐TrFE) 65/35 mol%] and relaxor terpolymer [P(VDF‐TrFE‐CFE) 61.5/30.3/8.2 mol% (CFE: chlorofluoroethylene)] as different components to form a blend. Copolymer is in the all‐*trans* conformation while it was assumed that relaxor terpolymer takes a disordered 3/1 helical conformation just like relaxor copolymers. Since both polymers do not cocrystallize,^163^ a mixture of the *trans*‐planar and 3/1‐helical phases can be achieved (Figure 8b). As a result, the change in the volume ratio between the copolymer and terpolymer, i.e., Co/Ter would lead to tuning of the faction of 3/1‐helical phase.

**Figure 9:**
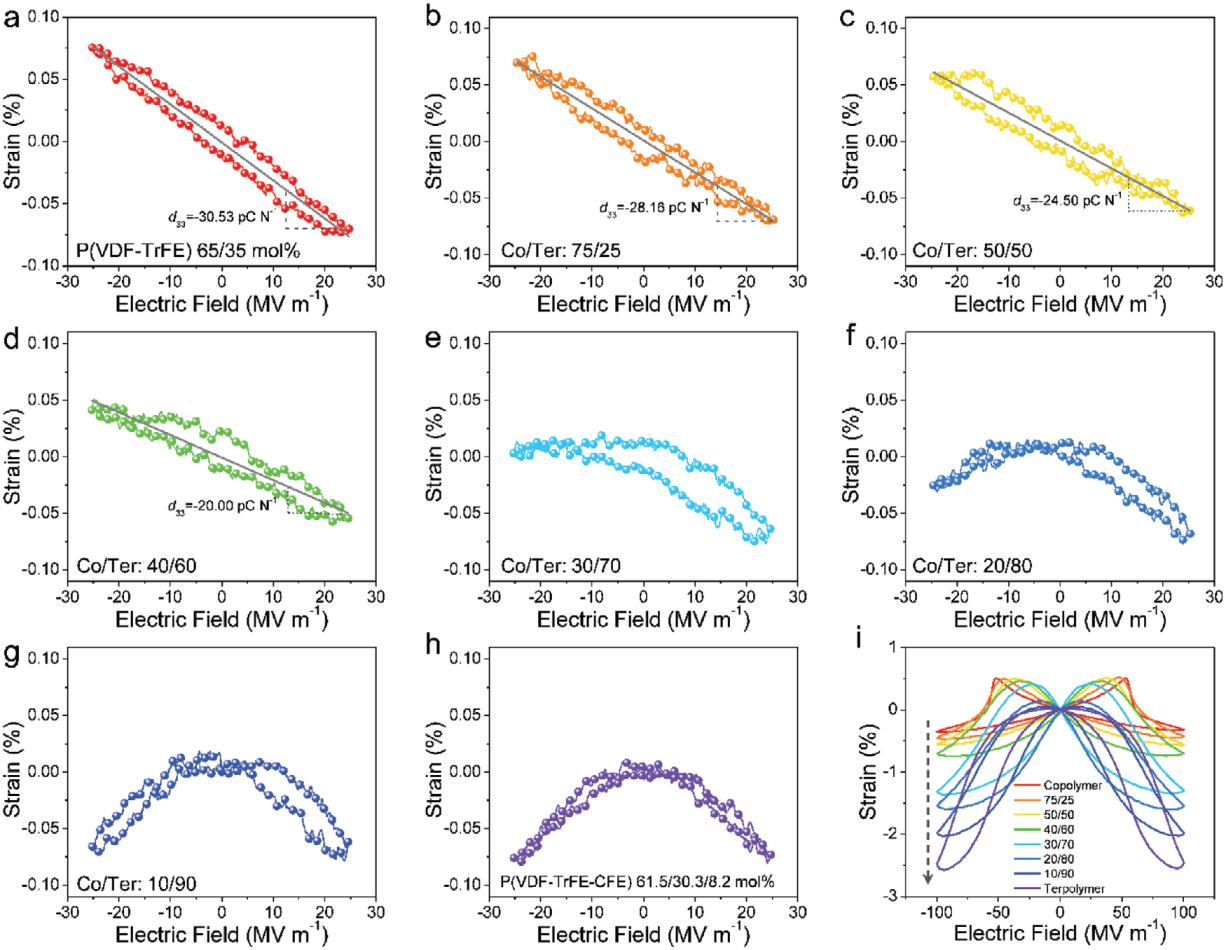
a–i) Electric‐field‐induced strain in the blends with various compositions, measured by a 1 Hz triangular waveform of a bipolar electric field at room temperature. The blend corresponds to a mixture of ferroelectric copolymer P(VDF‐TrFE) 65/35 mol% and relaxor terpolymer P(VDF‐TrFE‐CFE) 61.5/30.3/8.2 mol%. The dashed line in (i) indicates the strain enhancement with increasing the terpolymer content. Adapted with permission.^164^ Copyright 2019, American Chemical Society.

The experimental results^164^ shown in **Figure** **9** may disregard the proposed case in Figure 8b because a simple evolution from piezoelectric linear curve (Co/Ter: 100/0, Figure 9a) to electrostrictive parabolic type (Co/Ter: 0/100, Figure 9h) was found as the terpolymer fraction increases. There is no evidence of enhanced piezoelectric responses inherent to the existence of MPB.^51^, ^52^, ^132^, ^133^, ^134^, ^135^, ^136^, ^138^, ^139^ The longitudinal piezoelectric coefficient *d*_33_ deduced from the slope of strain–field curves shows a gradual reduction, as the terpolymer fraction increases (Figure 9a–d). The electrostriction is dominant above a critical terpolymer content of 70% (Co/Ter: 30/70, Figure 9e) and the strain sign does not change as the field direction is altered. After that, the strain curve becomes more symmetric and the piezoelectric contribution can be negligible. Such strain evolution bears a resemblance to temperature‐driven phase transition from ferroelectric to paraelectric phase, provided that relaxors at small electric fields cannot have piezoelectricity because they are macroscopically paraelectric. The strain–field responses at high electric fields show further evidence of the absence of MPB in the blends (Figure 9i).

On the basis of the single chain model, the structural parameters such as length of polymer chain and polydispersity can also significantly affect the physical properties of polymers. Comparison of the NMR spectra and piezoelectric properties between Piezoetch Arkema and Solvey and the home‐made P(VDF‐TrFE) were carefully done.^50^ Almost no difference has been identified in terms of the chemical microstructures and piezoelectric responses.^50^ Specifically, it is found that the ^1^H and ^19^F NMR spectra of the commercial samples are almost identical to those of the polymer synthesized in the lab, indicating that they have nearly the same regiosequences and stereosequences. The commercial samples possess only slightly lower contents of regiodefect (H‐H/T‐T) than the synthesized polymer and nearly the same distributions of chain tacticity as the synthesized P(VDF‐TrFE). Importantly, all three P(VDF‐TrFE) (65/35 mol%) films display the same *d*_33_ value of ≈−30 pC N^−1^. These findings prove that the MPB formation is not due to any uniqueness that might only exist in the home‐made P(VDF‐TrFE) while it is actually a general phenomenon only related to the polymer composition.

### 3.6 Mechanisms of the MPB Behavior in P(VDF‐TrFE)s

**Figure 10:**
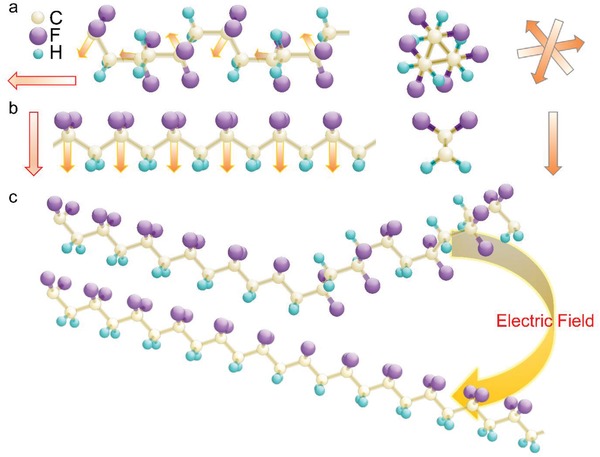
a,b) Schematic of 3/1‐helical and *trans*‐planar conformations. The orange arrows correspond to the projections of the —CF_2_ dipole directions on planes defined by the CF_2_ groups. The red arrows indicate the net polarization direction. The right panels are the side views of 3/1‐helical and all‐*trans* conformations where the arrows indicate the directions of in‐plane polarization. c) Schematic of electric‐field‐induced conformation change.

Polarization rotation mechanism in terms of the maximized flexibility of polarization rotation between different symmetries is regarded as the most widely used concept to understand enhanced piezoelectric properties observed at MPB in perovskite ceramics.^51^, ^135^ There are other mechanisms such as the polarization extension,^165^, ^166^, ^167^ twined nanodomains,^168^, ^169^, ^170^, ^171^ domain wall contributions,^172^, ^173^ grain size effect,^174^ and so on, which may also explain the enhanced piezoelectric response near MPB. In the case of P(VDF‐TrFE), the flattening of the energy landscape arising from the conformational competition between the *trans*‐planar and 3/1‐helical type phases leads to formation of MPB, according to the first‐principles calculations.^50^ The 3/1‐helical conformation possesses the net polarization direction along the chain axis (**Figure** **10**a) while the net polarization in the all‐*trans* conformation is normal to the chain axis (Figure 10b).^52^, ^164^ It was suggested that the nearly vanishing barrier might maximize the flexibility of polarization rotation between the two phases and thus enhance the piezoelectric properties (Figure 10c).^52^, ^164^ MPB effect due to the polarization rotation may lead to enhanced piezoelectric shear strain.^175^ Further experiments^176^ are therefore highly desired not mentioning that large shear strain is of importance to devices of applications.^177^ Moreover, resolving the origins of enhanced piezoelectric properties in ferroelectrics should be cautious as many foregoing complex factors may simultaneously contribute to strain response to external field.^178^

Obviously, it highly demands further developments especially from the aspect of ab initio calculations.^47^, ^160^, ^179^, ^180^ However, there are very limited theoretical studies on P(VDF‐TrFE)s using the ab initio methods. Very few experimental results on the TrFE‐rich P(VDF‐TrFE)s significantly hamper the theoretical efforts. In most of the current models the role of chain tacticity has been, unfortunately, overlooked. For instance, most studies focus on quantifying or reproducing the ferroelectric properties of PVDF and P(VDF‐TrFE)s with the assumption that the all‐*trans* conformation is the ground state of the polymers.^150^, ^161^, ^181^, ^182^ A more recent density functional theory study ignored the contributions of chain tacticity and showed that all‐*trans* conformation is the most energetically favorable for both P(VDF‐TrFE)s and PTrFE,^162^ which is in contrast to recent experimental results on P(VDF‐TrFE)s^50^ and PTrFE^183^, ^184^ as well as the previous conformational energy calculations on PTrFE.^54^ The computational studies of P(VDF‐TrFE)s provide a detailed energy difference between the configurations that are formed due to the chirality (isotactic/syndiotactic) and *trans*‐*gauche* effects (all‐*trans* and 3/1 helix) among the copolymers with different VDF contents and predict the region where the MPB occurs, which is consistent with the experiment results.^50^ This striking contrast indicates that chain tacticity is critical to determine the ground state of P(VDF‐TrFE)s.

Although the underlying mechanism of MPB formation in P(VDF‐TrFE) demands further investigations, the discovery of MPB opens a new route to design high‐performance piezoelectric polymers. Before that, previous approaches to enhance piezoelectric properties of PVDF and its copolymers mainly focus on the enhancement of the β phase content.^12^, ^35^ In this regard, the fundamental implication of the discovery of MPB‐like behavior in P(VDF‐TrFE) copolymer is important, as it overturns this common belief^12^ showing that instability of the β phase can be utilized to significantly improve the piezoelectric performances. The reduction of the β phase fraction of P(VDF‐TrFE) copolymers is achieved by the growth of the competing 3/1‐helical phase, which is accompanied by the large smearing of the energy barrier between these two structures.^50^ Given that PVDF and its copolymers exhibit a rich family of crystalline phases with a relative low energy barrier,^6^, ^146^ the interconversion between different structures can be induced by various methods including stress,^185^ pressure,^186^ electric field,^187^ grafting,^188^, ^189^ defects,^127^, ^128^, ^190^ irradiation,^126^, ^191^, ^192^, ^193^ and so on. However, there remains lack of piezoelectric data associated with the transition. It is therefore sausible that the enhancement of piezoelectric responses during the phase transition may be anticipated, because the strain and pressure have been used to drive MPB and thus significantly improved piezoelectric effect in perovskite ferroelectrics.^135^, ^136^

## 4 Conclusions and Perspectives

In summary, we have reviewed different theoretical models to understand the negative longitudinal piezoelectric coefficient in ferroelectric polymers. In particular, we discuss the crucial role of the origin of this issue in the discovery of the MPB in P(VDF‐TrFE) copolymers. We have determined electrostrictive coefficients of P(VDF‐TrFE)s with MPB compositions based on the electrostrictive measurements and revealed that the negative longitudinal piezoelectric coefficient originates from the longitudinal electrostriction in the crystalline domain of P(VDF‐TrFE)s. Moreover, it is believed that MPB shall be a general phenomenon especially in ferroelectric polymers with multiple single‐chain crystalline conformations, which will stimulate further search for new MPB piezoelectric polymers and offers unparalleled possibilities to enhance the piezoelectric properties of polymers.

To rapidly search for potential MPB polymers with greatly improved electromechanical responses, it requires that a candidate polymer exhibits a rich crystalline phase diagram, as defined by its crystalline chain conformations. A rotation of covalent bonds could lead to interconversion between conformational isomers, which could generate two nearly energetically degenerate phases. In most cases, different crystalline phases correspond to different crystallographic structures in ferroelectric polymers, which naturally meets the requirement of symmetry breaking between different crystalline phases.

The crystalline origin of negative longitudinal piezoelectric coefficient is of great fundamental importance. On the one hand, it invites first‐principles calculations research aiming to provide deeper insights into changes of lattice or molecular structures in response to an applied electric field, which would enable tailoring a variety of physical properties, such as dielectric constants and electromechanical coupling coefficients, of polymers through structural engineering, just like their inorganic counterparts. On the other hand, it introduces open‐ended questions that inspire scientific thinking in totally new directions, such as search of novel MPB piezoelectric polymers, polarization rotation mechanisms, and activation of the amorphous domains to be piezoelectric active. It is believed that the research in this emerging area would not only broaden the well‐established concept in inorganic piezoelectric materials but also open up new perspectives for developing high‐performance piezoelectric polymers for next‐generation flexible, wearable, and biocompatible applications.

The research in the field of MPB piezoelectric polymers is at its infancy stage, e.g., the development of relevant theoretical models with predictive capability is urgently needed. New theoretical concepts need to be developed in order to understand the complex phase behaviors and piezoelectric responses of ferroelectric polymers and underlying mechanisms of molecular‐level MPB. Novel synthesis methods are needed to precisely control the chain conformation, tacticity, and crystalline phases of ferroelectric polymers as guided by predictive multiscale modeling. Machine learning and data mining can be utilized to elucidate and predict the roles of polymer structure and composition in the MPB formation and piezoelectric properties of polymers. As a highly interdisciplinary field, progress in piezoelectric polymers is critically dependent on successful interactions across the boundaries of traditional disciplines. Rapid advances are to be expected through collaborative efforts from synthetic chemists, physicists, and theorists.

## Conflict of Interest

The authors declare no conflict of interest.
